# Comparative Analysis of African Swine Fever Virus Genotypes and Serogroups

**DOI:** 10.3201/eid2102.140649

**Published:** 2015-02

**Authors:** Alexander Malogolovkin, Galina Burmakina, Ilya Titov, Alexey Sereda, Andrey Gogin, Elena Baryshnikova, Denis Kolbasov

**Affiliations:** National Research Institute for Veterinary Virology and Microbiology, Russian Academy of Agriculture Science, Pokrov, Russia

**Keywords:** African swine fever virus, genotypes, phylogenetic analysis, serogroups, hemadsorption inhibition assay, viruses, vaccine development, swine, domestic pigs, cross-protective responses, antigenic diversity

## Abstract

African swine fever virus (ASFV) causes highly lethal hemorrhagic disease among pigs, and ASFV’s extreme antigenic diversity hinders vaccine development. We show that p72 ASFV phylogenetic analysis does not accurately define ASFV hemadsorption inhibition assay serogroups. Thus, conventional ASFV genotyping cannot discriminate between viruses of different virulence or predict efficacy of a specific ASFV vaccine.

African swine fever (ASF) is a highly contagious hemorrhagic disease that causes high rates of death among domestic pigs. The disease is caused by ASF virus (ASFV), and the extreme antigenic diversity of the virus is one of the main obstacles to developing a safe and efficacious vaccine against ASF. Nevertheless, substantial progress has been made in understanding the pathogenesis of the disease and virus–host interactions (*1*,*2*). The ability to induce a protective immune response against ASFV has been demonstrated in numerous studies. Pigs that recover from ASF have long-term immunity to subsequent challenge with moderately virulent ASFV and related virulent viruses, but they rarely gain immunity to heterologous viruses (*3*–*6*). Because of these cross-protective responses, the antigenic diversity among naturally occurring ASFV isolates is of interest for ASFV vaccine development (*7*).

Researchers at the National Research Institute for Veterinary Virology and Microbiology (VNIIVViM) in Рokrov, Russia, have developed a classification of ASFV isolates based on a hemadsorption inhibition assay (HAI) with ASFV reference immune antisera. The results of a long-term study from VNIIVViM were used to serologically classify ASFV strains, isolates, and attenuated variants. Eight serogroups have been identified (serogroups 1–8), but more likely exist. In vaccine design and development, consideration should be given to the fact that viruses within a serogroup provide cross-protection from challenge with viruses of the same serogroup (*8*,*9*). VNIIVViM maintains a large and diverse collection of serologically grouped ASFV isolates that provides a unique resource for defining ASFV strain variability and establishing relationships of cross-protective immunity (*10*,*11*).

Current genetic typing of ASFV isolates is based on nucleotide sequencing of the p72 capsid protein gene (B646L) and/or amplification of full-length polymorphisms of various genomic regions (*12*,*13*). During ASF outbreaks, these genotyping approaches can be used to identify the origin of viruses and quickly differentiate closely related strains. However, the correlation between currently established ASFV genotypes and viral cross-protection is not precisely clear (*6*). Thus, we examined the relationship of the established genotype distribution to HAI serologic classification.

## The Study

Serologic classification was based on HAI results for ASFV strains maintained at VNIIVViM. These include isolates from disease outbreaks in Africa, Europe, the Caribbean, and, more recently, from the Russian/Trans-Caucasian epizootic and attenuated variants. In brief, swine red bone marrow cell culture was used for ASFV isolate amplification, and swine anti-ASFV serum and erythrocytes were subsequently added to the culture. ASFV isolates for which the hemadsorption phenomenon was inhibited by serum belonging to the same group within serogroups 1–8 were clustered into a homologous serogroup. Only ASFV hemadsorbing strains could be analyzed by this method, and some hemadsorbing ASFV isolates could not be placed into existing serogroups because HAI was not observed with available reference serum (*8*,*9*).

ASFV isolates from the depository at VNIIVViM were also classified by using a standard ASFV genotyping protocol previously published by Bastos et al. (*13*). In this method, the variable part of the p72 (B646L) gene was amplified by conventional PCR, and the amplicons were directly sequenced by using a 3130xl Genetic Analyzer (Applied Biosystems, Foster City, CA, USA) according to manufacturer’s recommendations. Chromatograms were manually edited and assembled by using CAP3 (http://pbil.univ-lyon1.fr/cap3.php). The nucleotide sequences of the ASFV isolates were deposited into GenBank (accession nos. KJ526354–KJ5263471).

**Figure 1:**
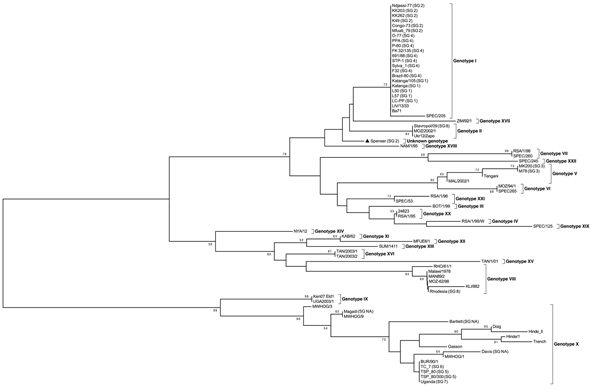
Phylogenetic tree of African swine fever virus (ASFV) isolates maintained in a collection at the National Research Institute for Veterinary Virology and Microbiology in Рokrov, Russia; the variable part of B646L gene relative to the 22 known p72 genotypes (labeled I-XXII) was used for analysis. The tree was reconstructed by using the minimum evolution method with 1,000 replicates.

Sequences determined at VNIIVViM were aligned with other publically available ASFV sequences and analyzed by using minimum evolution; a rooted tree was constructed with MEGA 5.0 software (*14*) and edited with FigTree v1.4 (http://tree.bio.ed.ac.uk/) (Figure 1). The results of the ASFV genotyping are summarized in the Table.

**Table:** ASFV isolates selected for inclusion in a study comparing ASFV genotypes and serogroups*

Isolate	Country of origin†	Year			p72 genotype	GenBank accession no.	Serogroup
		Isolated from primary outbreak	Isolate deposited‡	Attenuated variant deposited‡			
L57	Portugal	1957	1982	–	I	AF301537.1	1
L50	Portugal	NK	1983	–	I	AF301537.1	1
LC-PP	Portugal	–	1967	1967	I	AF301537.1	1
Katanga	Zaire (DRC)	NK	1978	–	I	KJ526355	1
Katanga/105	Zaire (DRC)	NK	1978	–	I	KJ671546	1
STP-1	Sao-Tome and Principe	1979	1979	–	I	KJ526371	4
P-60	Portugal	NK	1978	–	I	AF301539	4
F-32	France	1964	1969	–	I	KJ671547	4
FK-32/135	France	–	1973	1973	I	KJ526370	4
О-77	USSR (Ukraine)	1977	1977	–	I	KJ671544	4
Brasil-80	Brazil	1979	1980	–	I	KJ526367	4
691/88	Switzerland	NK	1989	–	I	KJ671549	4
PPА	Spain	NK	1984	–	I	KJ526362	4
КК262	Zaire (DRC)	–	1989	1992	I	KJ526364	2
КК202	Zaire (DRC)	–	1974	1974	I	KJ526363	2
К49	Zaire (DRC)	1949	1983	–	I	KJ671543	2
Ndjassi-77	Zaire (DRC)	1977	1979	–	I	KM236553	2
Sylva 1	Angola	1982	1982	–	I	KJ526365	2
Mfuati-79	Congo (People’s Republic of Congo)	1979	1980	–	I	KJ526368	2
Congo-73	Zaire (DRC)	NK	1983	–	I	KJ671545	2
М78	Mozambique	NK	1978	–	V	KJ671548	3
МК200	Mozambique	–	1980	1980	V	KJ526369	3
Stavropol 01/08	Russia	2008	2009	–	II	JQ771686	8
TSP 80	Tanzania	NK	1967	–	X	KJ526361	5
TSP80/300	Tanzania	–	1986	1986	X	KJ526366	5
Bartlett	Kenya	NK	1961	–	X§	KJ526356	ND
Uganda	Uganda	NK	1984	–	X	KJ526359	7
Magadi	Kenya	NK	1984	–	X§	KJ526358	ND
Davis	Кenya	NK	1986	–	X§	KJ526357	ND
TS-7	Tanzania	NK	1967	–	X	KJ526360	6
Rhodesia	Rhodesia (Zimbabwe)	NK	1986	–	VIII	KJ671542	8
Spenser	Republic of South Africa	NK	1985	–	?¶	KJ526354	2

*The isolates were selected from the African swine fever virus (ASFV) collection at the National Research Institute for Veterinary Virology and Microbiology (VNIIVViM) in Рokrov, Russia. ASFV genotypes are assigned according to p72 (B646L) nucleotide sequencing and phylogenetic tree reconstruction. Serogroups are defined on the basis of results of hemadsorption inhibition assay (HAI) with reference serum (serogroups 1–8). ASFV isolates from primary outbreaks as well as attenuated variants have been characterized. Democratic Republic of Congo; –, not applicable; NK, not known; ND, not defined. †If a country name changed since the virus was isolated, the current name of the country is shown in parentheses. ‡Deposited into the collection at VNIIVViM. §Isolates Bartlett, Magadi, and Davis could not be placed into an existing serogroup because no HAI was observed with available reference serum, so exact serogroups for these isolates are not defined. ¶Isolate Spenser does not belong to any of the 22 known genotypes and remains untyped.

**Figure 2:**
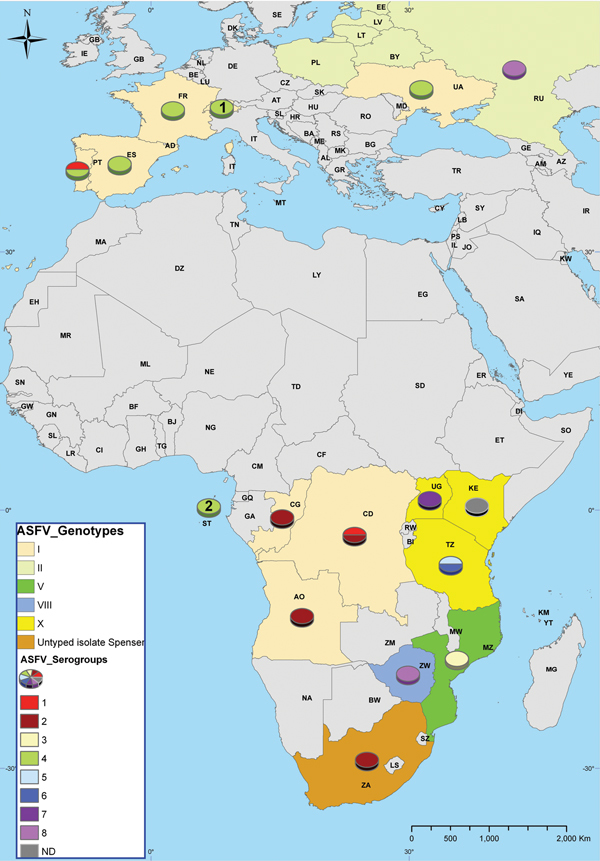
World distribution of African swine fever virus (ASFV) isolates maintained in a collection at the National Research Institute for Veterinary Virology and Microbiology in Рokrov, Russia. Results of p72 genotyping and hemadsorption inhibition assay of ASFV isolates are summarized on the map. Genotype II of ASFV isolates from Lithuania, Latvia, Estonia, Poland, and Belarus was identified by CISA-INIA (Animal Health Research Center; European Union Reference Laboratory for African Swine Fever). ASFV isolate O-77, which was isolated in 1977 from Odessa, Ukraine (at the time, part of the Union of Soviet Socialist Republics), was used in this study. On the basis of CISA-INIA results, currently circulating isolates in Ukraine belong to genotype II. ASFV isolate Brazil-80 (genotype I, serogroup 4) is not shown. The oval with a 1 inside indicates Switzerland; the oval with a 2 inside indicates São Tome and Principe. Country names are presented as 2-letter country codes as designated by the International Organization for Standardization country codes (ISO 3166, http://www.iso.org/iso/country_codes.htm).

Newly identified ASFV genotypes and known serogroups were mapped together so their geographic distribution in Africa and Europe (including the European part of the Russian Federation) could be visualized. The results (Figure 2) show that genotypic and serogroup diversity are greatest in a relatively limited area, mainly in southeastern Africa. In contrast, non-ASFV–endemic countries, where ASF outbreaks were caused by ASFV of a single genotype, exhibited low or no serogroup diversity.

Single genotype clades of ASFV were observed to contain viruses of multiple serogroups (Table). For example, ASFV isolates belonging to serogroups 1, 2, and 4 were specifically clustered within genotype I, and did not group with other genotypes. This indicates heterogeneity among ASFV strains previously isolated on the European continent.

We also found several serogroups of ASF viruses within genotype X. The ASFV isolates TSP80 (serogroup 5) and TS-7 (serogroup 6) were subsequently isolated from 1 field sample derived from a naturally infected pig in Tanzania. However both were genotype X viruses. Of note, 1 serogroup 2 isolate (Spenser) demonstrated a novel genotype within the p72 phylogenetic tree and relative to other serogroup 2 viruses (Figure 1), indicating that the p72 genotype, in addition to lacking serotype resolution, has potential to be incongruous relative to serogroup. Together, these data indicate that the antigenic heterogeneity of ASFV strains is not fully captured by using the standard genotyping approach.

## Conclusions

The virus elements responsible for protective and cross-protective immune responses are not well known. Given the structural and genetic complexity of ASFV, it is likely that genes encoding different antigens will be more suited for virus typing. Substantial genetic variability can exist between strains and predominate in specific genomic regions, and it is these regions that may provide improved targets for genotyping. Our findings support that of a previous study that showed that geographic areas with ASFV of high genotypic and serotypic diversity are located in countries where multiple mechanisms of ASF transmission (mixed sylvatic and domestic cycle) are established (*11*).

HAI serology provides a measure of ASFV typing that, compared with p72 genotyping, better discriminates biologically pertinent phenotypes. Viruses belonging to one p72 genotype may be serotypically heterogeneous: strains that are closely related genetically, even from a single isolate, may have different phenotypes and form homologous serogroups. Our assessment of ASFV genotyping relating to HAI serotyping shows the serologic diversity within a p72 genotype. Our results highlight the potential for using serogroup classification to understand issues of homologous cross-protection among ASFV isolates and virus determinants that influence disease emergence.

The key finding from our study is that p72 ASFV phylogenetic analysis fails to accurately define ASFV HAI serogroups. Thus, conventional ASFV genotyping cannot discriminate between viruses of different virulence or predict the efficacy of a specific ASFV vaccine. This finding also suggests that for vaccination-based control of ASF, it may be more important to determine serogroups rather than genotypes of ASFV isolates. Nevertheless, more ASFV sampling is needed to further define genotypes and serogroups.
